# Supplementary material for: The TDRD3-USP9X complex and MIB1 regulate TOP3B homeostasis and prevent deleterious TOP3B cleavage complexes
Source: Nat Commun. 2023 Nov 18;14:7524. doi: 10.1038/s41467-023-43151-z (PMC10657456; doi:10.1038/s41467-023-43151-z)
Supplement: Supplementary file 1 — Supplementary Information [file 41467_2023_43151_MOESM1_ESM.pdf]

Supplementary Information for

The TDRD3-USP9X complex and MIB1 regulate TOP3B homeostasis and prevent deleterious TOP3B cleavage complexes

Authors

Sourav Saha[1], Shar-yin Naomi Huang[1], Xi Yang[1], Liton Kumar Saha[1], Yilun Sun[1], Prashant Khandagale[1], Lisa M. Jenkins[2], and Yves Pommier[1*]

Affiliations

[1]Developmental Therapeutics Branch & Laboratory of Molecular Pharmacology, Center for Cancer Research, National Cancer Institute, NIH, Bethesda, MD 20892, USA
[2]Collaborative Protein Technology Resource, Center for Cancer Research, National Cancer Institute, NIH, Bethesda, MD 20892, USA

[*]**Lead Contact and Corresponding Author.** Email: pommier@nih.gov Tel: 240-760-6142; Fax: 240-541-4475

This PDF file includes:

Supplementary Figures 1-7

Supplementary Figure Legend 1-7

Supplementary Tables 1-2

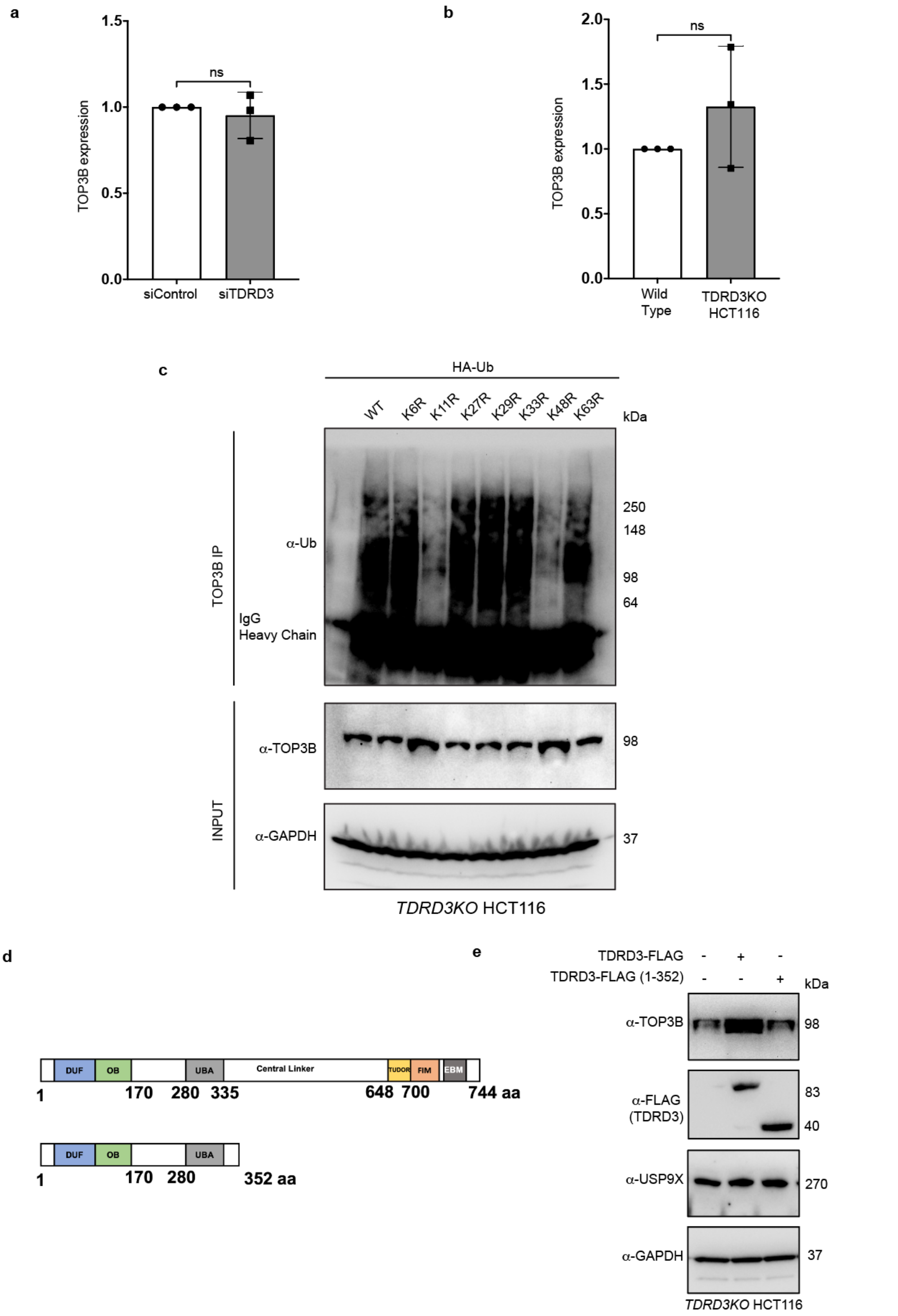

Supplementary Figure 1

Supplementary Fig. 1: TDRD3 protects TOP3B from ubiquitylation and proteasomal targeting. (Related to Fig. 1 and Fig. 2)

a. Quantitative real-time reverse transcription PCR (qRT-PCR) results for *TOP3B* gene expression in control and *TDRD3* siRNA transfected HEK293 cells. Each transcript was normalized to the expression of Actin. Results are the average of three independent experiments with ± SD (n=3). 'ns': not significant (two-tailed paired t test).

b. qRT-PCR for *TOP3B* gene expression in wild type and *TDRD3KO* HCT116 cells. Each transcript was normalized to the expression Actin. Results are the average of three independent experiments with ± SD (n=3). 'ns': not significant (two-tailed paired t test).

c. Free cellular TOP3B ubiquitylation in the absence of TDRD3 involves proteasomal-specific linkages to lysines K11 and K48. *TDRD3KO* HCT116 cells transfected either with wild-type HA-tagged ubiquitin (Ub) or HA-tagged lysine-to-arginine Ub mutants for each of the 7 lysine residues (K6R-Ub, K11R-Ub, K27R-Ub, K29R-Ub, K33R-Ub, K48R-Ub, and K63R-Ub) and subjected to TOP3B pulldown. Pulled-down and input samples were resolved on SDS-PAGE and probed with Ubiquitin and TOP3B antibodies. Experiment performed three times independently with similar results .

d. Schematic representation of the domain organization of human TDRD31 (1-744 aa) and C-terminal deletion mutant of TDRD3 (1-352 TDRD3) that cannot interact with USP9X.

e. Ectopic expression of the C-terminal deletion mutant of TDRD3 (1-352 TDRD3) does not stabilize TOP3B. *TDRD3KO* HCT116 cells were transfected with the full-length and 1-352 TDRD3-FLAG constructs and incubated for 48 h. Cell lysates were subjected to Western blotting with TOP3B, USP9X and TDRD3 antibodies (GAPDH as loading control). Experiments performed three times independently with similar results.

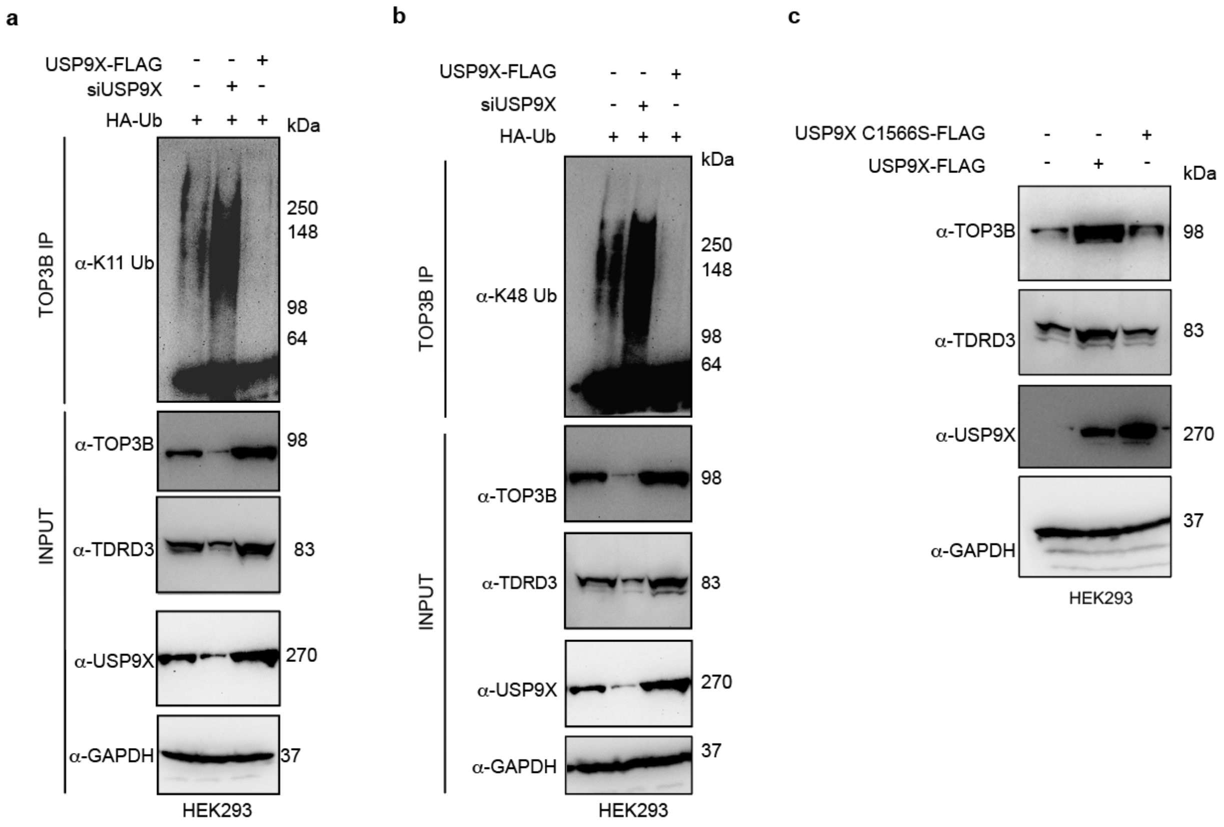

Supplementary Figure 2

Supplementary Fig. 2: The deubiquitylation activity USP9X is important for removing K11- and K48-linked ubiquitin chains from TOP3B and subsequent stabilization. (Related to Fig. 2 and Fig. 3)

a-b. USP9X removes both K11- and K48-linked ubiquitin chains from TOP3B. HEK293 cells were transfected with the HA-Ubiquitin construct and siUSP9X or USP9X-FLAG (as indicated) for 48 h and subjected to TOP3B pulldown. Pulled-down and input samples were resolved on SDS-PAGE and probed with K11- or K48-specific ubiquitin, TOP3B, TDRD3 and USP9X antibodies (GAPDH as loading control for input samples). Experiments performed three times independently with similar results.

c. The deubiquitylation activity of USP9X is important for stabilizing TOP3B and TDRD3 proteins. After transfection of HEK293 cells with either the wild-type USP9X-FLAG or catalytically inactive USP9X (C1566S USP9X-FLAG) construct for 48 h, cell lysates were subjected to Western blotting with TOP3B, TDRD3 and USP9X antibodies (GAPDH as loading control). Experiments performed three times independently with similar results.

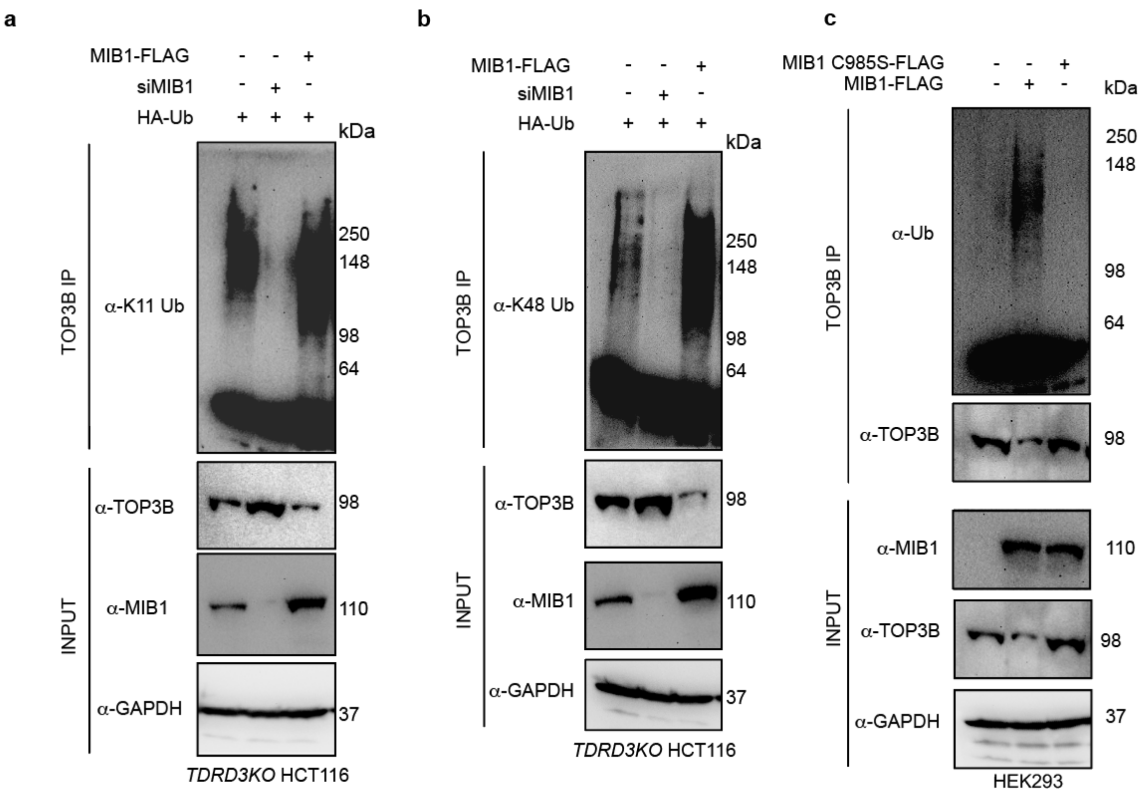

Supplementary Figure 3

Supplementary Fig. 3: The E3 ubiquitin ligase activity of MIB1 is important for K11- and K48-linked polyubiquitin chain formation on TOP3B and proteasomal degradation. (Related to Fig. 4)

a-b. MIB1 can add both K11- and K48-linked ubiquitin chains on free TOP3B in the absence of TDRD3. *TDRD3KO* HCT116 cells were transfected with the HA-Ubiquitin construct and siMIB1 or MIB1-FLAG (as indicated) for 48 h and subjected to TOP3B pulldown. Pulled-down and input samples were resolved on SDS-PAGE and probed with K11- or K48-specific ubiquitin, TOP3B, TDRD3 and MIB1 antibodies (GAPDH as loading control for input samples). Experiments performed three times independently with similar results.

c. The E3 ubiquitin ligase activity of MIB1 is necessary for the ubiquitylation and subsequent proteasomal degradation of TOP3B. After transfection of HEK293 cells with either the wild-type MIB1-FLAG or catalytically inactive MIB1 (C985S MIB1-FLAG) constructs for 48 h, cell lysates were subjected to Western blotting with ubiquitin, TOP3B, and MIB1 antibodies (GAPDH as loading control). Experiments performed three times independently with similar results.

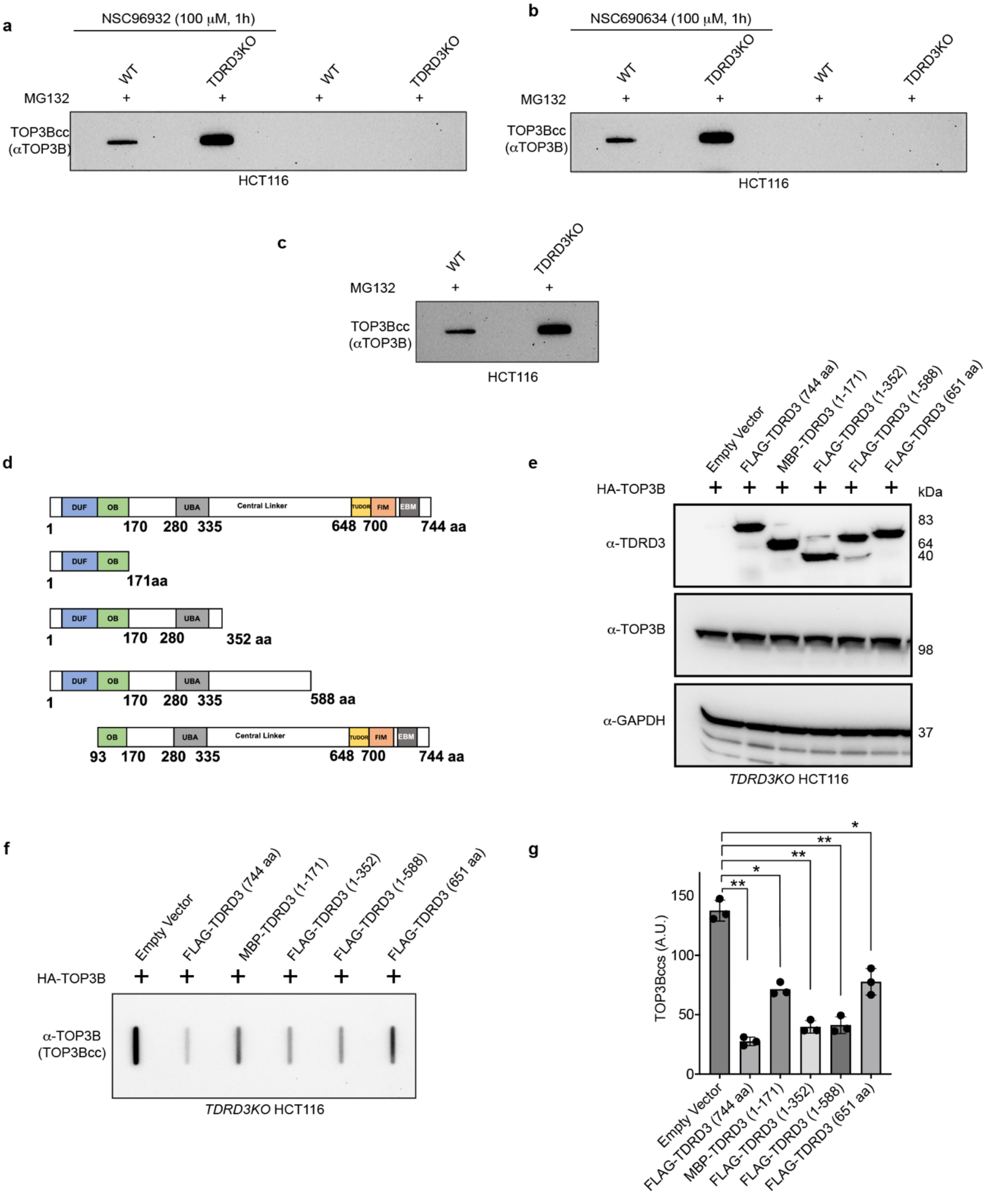

Supplementary Figure 4

Supplementary Fig. 4: TDRD3 prevents the accumulation of endogenous TOP3B DNA and RNA cleavage complexes (TOP3Bccs) and the N-terminus of TDRD3 is important for regulating cellular TOP3Bcc levels. (Related to Fig. 5)

a-b. Accumulation of endogenous TOP3Bccs in *TDRD3KO* HCT116 cells following proteasome inhibition and treatment with the TOP3B trapping drugs NSC690634 and NSC96932. RADAR assay samples were prepared from wild-type and *TDRD3KO* HCT116 cells treated with MG132 (10 μM, 3 h) and NSC96932 (100 μM, 1 h) or NSC690634 (100 μM, 1 h). Experiments performed three times independently with similar results.

c. Accumulation of TOP3Bccs in *TDRD3KO* HCT116 cells following proteasomal inhibition and IP enrichment of TOP3Bccs. RADAR assay samples were prepared from MG132-treated wild-type and *TDRD3KO* HCT116 cells, immunoprecipitated with TOP3B antibody, eluted from the beads, ethanol precipitated and resuspended. Immunoprecipitated RADAR assay samples were slot blotted and TOP3Bccs were detected. Experiments performed three times independently with similar results.

d. Schematic representation of the domain organization of human full length TDRD3 (1-744 aa), C-terminal deletion mutants of TDRD3 (1-171 TDRD3, 1-352 TDRD3 and 1-588 TDRD3), TDRD3 transcript isoform 2 (651 aa).

e. Western blot showing expression levels of different TDRD3 variants, TOP3B and GAPDH. *TDRD3KO* HCT116 cells co-transfected with HA-TOP3B and/or five different TDRD3 variants. Experiments performed three times independently with similar results.

f-g. The N-terminus of TDRD3 regulates TOP3Bcc reversal in cells. RADAR assay samples were prepared from *TDRD3KO* HCT116 cells co-transfected with HA-TOP3B and/or different TDRD3 variants. Samples were immunoprecipitated with TOP3B antibody, slot blotted and TOP3Bccs were detected with TOP3B antibody. Panel f displays a representative slot blot. Panel g is the quantitation of TOP3Bcc formation from three independent experiments. Data are means ± standard deviations (SD) (n = 3). ** P value = 0.0009 (Empty Vector vs FLAG-TDRD3 (744 aa)), * P value = 0.001 (Empty Vector vs MBP-TDRD3 (1-171 aa)), ** P value = 0.0005 (Empty Vector vs FLAG-TDRD3 (1-352 aa)), * P value = 0.0004 (Empty Vector vs FLAG-TDRD3 (1-588 aa)), ** P value = 0.005 (Empty Vector vs FLAG-TDRD3 (651 aa)) (two-tailed paired t test). A.U. is arbitrary units.

a

WT or *TDRD3KO* HCT116 cells

↓

IP with TOP3B Ab or IgG

↓

Mass Spec

	WT		*TDRD3KO*	
	IgG IP	TOP3B IP	IgG IP	TOP3B IP
TOP3B	0	83	0	84
PARP1	1	9	2	187
XRCC1	0	0	0	10
LIG3	0	0	0	34
TDRD3	0	42	0	0

b

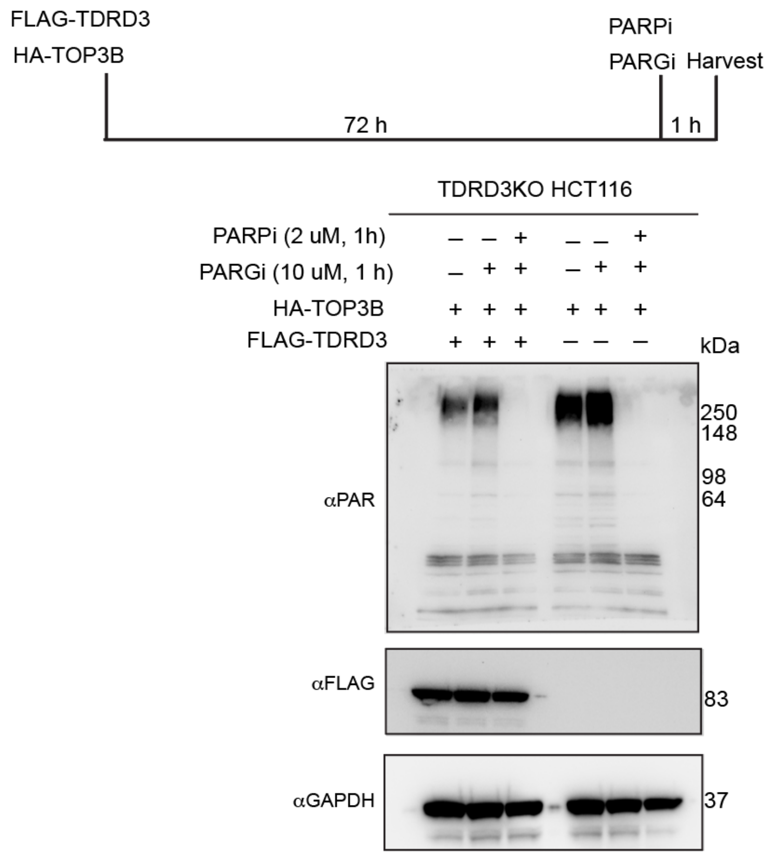

Supplementary Figure 5

Supplementary Fig. 5: TOP3Bcc formation activates PARP1 in TDRD3-deficient cells. (Related to Fig. 5)

a TOP3B pull-down-LC-MS/MS experiment showing enrichment of Base Excision/ Single Strand Break Repair proteins including PARP1 in *TDRD3KO* cells. Shown are the number of peptide-spectral matches (PSMs) identified.

b Increased PARP1 activity in *TDRD3KO* cells after TOP3Bcc formation. Representative Western blot of PAR levels and FLAG-TDRD3. *TDRD3KO* HCT116 cells were transfected with HA-TOP3B alone or together with TDRD3-FLAG constructs (as indicated) and incubated for 72 h. Before harvest cells were treated with talazoparib (2 μM, 1 h) or PARG inhibitor (10 μM, 1 h). Cell lysates were subjected to Western blotting and immunoblotted with PAR and FLAG antibody (GAPDH as loading control). Experiments performed three times independently with similar results.

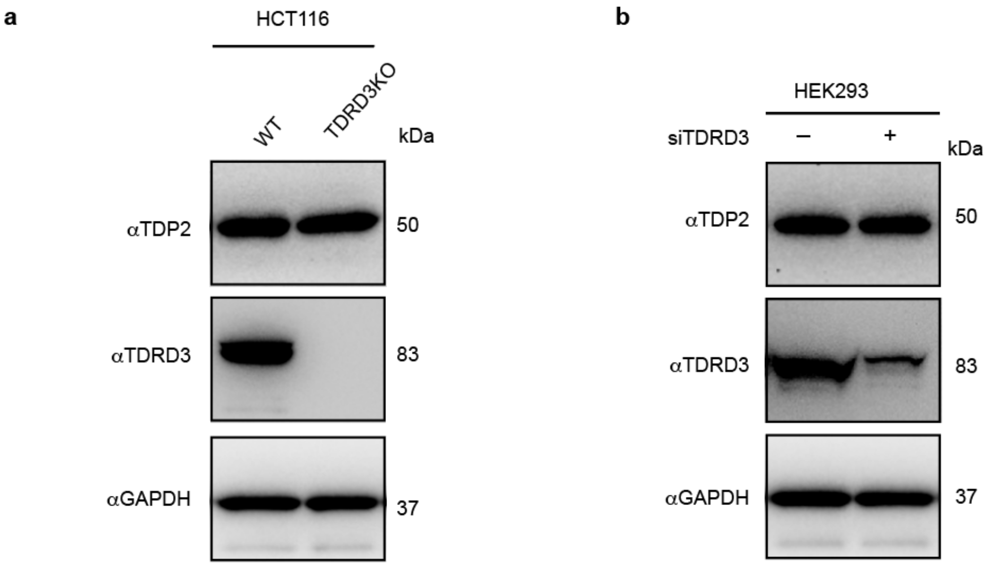

Supplementary Figure 6

Supplementary Fig. 6: TDRD3 depletion or accumulation of TOP3Bccs in the absence of TDRD3 do not change cellular TDP2 levels. (Related to Fig. 5)

a. TDRD3 depletion by siRNA for 48 h does not change TDP2 protein in HEK293 cells. Cell lysates were subjected to Western blotting and immunoblotted with TDP2 and TDRD3 antibodies (GAPDH as loading control). Experiments performed three times independently with similar results.

b. TDP2 expression levels in HCT116 wild-type and *TDRD3KO* cells. Cells were subjected to Western blotting with TDP2 and TDRD3 antibodies (GAPDH as loading control). Experiments performed three times independently with similar results.

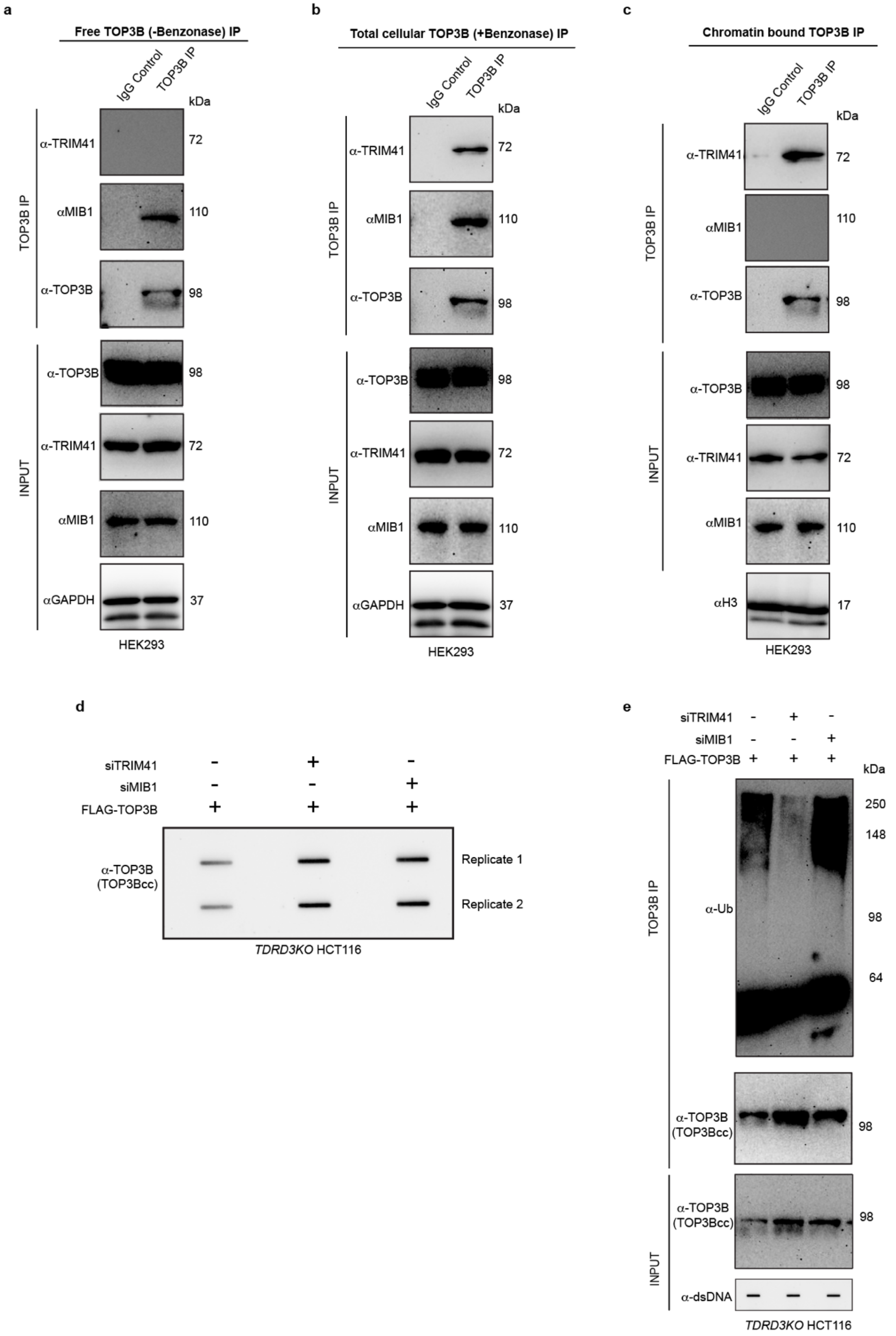

Supplementary Figure 7

Supplementary Fig. 7: MIB1 and TRIM41 act as designated E3 ligases for free TOP3B and TOP3B cleavage complexes, respectively. (Related to Fig. 4 and Fig. 5)

a. TOP3B pulldown-Western blot experiments showing that free TOP3B can only interact with MIB1. HEK293 cells were subjected to IgG or TOP3B IP using an immunoprecipitation (IP) buffer without benzonase. Experiments performed three times independently with similar results.

b. TOP3B pulldown-Western blot experiments showing that total cellular TOP3B (free and nucleic acids bound) interacts with both MIB1 and TRIM41. HEK293 cells were subjected to IgG or TOP3B IP using an immunoprecipitation buffer with benzonase. Experiments performed three times independently with similar results.

c. TOP3B pulldown-Western blot experiments showing that chromatin-bound TOP3B can only interact with TRIM41 and not MIB1. H3 was used as marker for chromatin fractions. Experiments performed three times independently with similar results.

d. Increased TOP3Bcc accumulation in the absence of both TRIM41 and MIB1. RADAR assay samples were prepared from *TDRD3KO* HCT116 cells transfected with FLAG-TOP3B alone or together with siTRIM41 or siMIB1 constructs (as indicated) for 48 h. Samples were immunoprecipitated with anti-TOP3B antibody, eluted from the beads, ethanol precipitated and resuspended. Samples were slot blotted and TOP3Bccs were detected with anti-TOP3B antibody. Experiments performed two times independently with similar results.

e. TRIM41 ubiquitylates TOP3Bccs in cells lacking TDRD3. RADAR assay samples were prepared from *TDRD3KO* HCT116 cells transfected with FLAG-TOP3B alone or together with siTRIM41 or siMIB1 constructs (as indicated) for 48 h and were immunoprecipitated with anti-TOP3B antibody. IP samples and the input RADAR assay samples were digested with micrococcal nuclease, resolved on SDS-PAGE, and immunoblotted with anti-Ub and anti-TOP3B antibodies. Loading of input RADAR samples was tested with anti-dsDNA antibody. Experiments performed three times independently with similar results.

Supplementary Table 1: List of Antibodies

Antibodies	Source	Catalogue Number
Monoclonal ANTI-FLAG® M2 Mouse Monoclonal antibody, Sigma-Aldrich	Millipore Sigma	Cat# F1804
Anti-GAPDH Rabbit Monoclonal Antibody, Unconjugated, Clone 14C10	Cell Signaling Technology	Cat# 2118S,
Anti-TOP3B Rabbit Monoclonal antibody [EP7779] - C-terminal (ab183520)	Abcam	Cat# ab183520
Rabbit Anti-HA-Tag Monoclonal Antibody, Unconjugated, Clone C29F4	Cell Signaling Technology	Cat# 3724S
Sheep Anti-Mouse IgG ECL Antibody, HRP Conjugated, GE Healthcare	GE Healthcare	Cat# NA9310-1ml
Donkey Anti-Rabbit IgG ECL Antibody, HRP Conjugated, GE Healthcare	GE Healthcare	Cat# NA9340-1ml
Mouse monoclonal anti-MIB1 antibody, Clone B9	Santa Cruz Biotechnology	CAT#: sc-393811
Anti-DNA-RNA Hybrid Antibody, clone S9.6	Millipore Sigma	Cat# MABE1095
Rabbit monoclonal anti-USP9X antibody	Cell Signaling Technology	CAT#: 14898S
Mouse monoclonal anti-phospho (S139)-H2AX (JBW301)	Millipore Sigma	CAT# 05-636
TDRD3 (D3O2G) Rabbit mAb #5942	Cell Signaling Technology	CAT# 5942S
Mouse monoclonal Anti-PAR Polymer Monoclonal Antibody	R&D Systems, Inc. a Bio-Techne Brand	CAT#: 4335-MC-100
Ub (P4D1) antibody	Santa Cruz Biotechnology	Cat# sc-8017
Rabbit polyclonal TRIM41 antibody	Abcam	Cat# ab111580

Supplementary Table 2: List of Primers

Primers	SOURCE
TOP3B forward primer for cloning into pcDNA3-HA: 5'- GCTTGGATCC AAG ACT GTG CTC ATG GTT-3'	IDT oligo
TOP3B reverse primer for cloning into pcDNA3-HA: 3'-CCAAGAATTCTCATACAAAGTAGGCGGC-5'	IDT oligo
TOP3B forward primer for cloning into vector pENTR3C: 5'-CGGGGTACCATGAAGACTGTGCTCATGG-3'	IDT oligo
TOP3B reverse primer for cloning into vector pENTR3C: 5'-AGGCTACATCAGCTACGTACGGACAGAGACCACC-3'	IDT oligo
TDRD3 forward primer for cloning into vector pDest-635: 5'-GGGGACAACTTTGTACAAAAAAGTTGGCGAAAACCTGTACTTCCAAGGCATGGCCCAGGTGGCCGGCGC-3'	IDT oligo
Hairpin DNA oligo substrate with long 3′-tail: GGGATTATTGAACTGTTGTTCAAACTTTAGAACTAGCCATCCGATTTACACTTTGCCCCTATCCACCCC-3'FITC	IDT oligo
Hairpin RNA oligo substrate with long 3′-tail: GGGAUUAUUGAACUGUUGUUCAAACUUUAGAACUAGCCAUCCGAUUUACACUUUGCCCCU-3' Cy5	IDT oligo
TOP3B qRT-PCR primers: TOP3B forward primer- 5'-GATGCTGGAGAAGCAGACGAAC-3', TOP3B reverse primer- 5'-CTCTCCACCGTGACATAGTTGC-3	IDT oligo
Actin qRT-PCR primers: Actin forward primer- 5'-TGCTATGTTGCCCTAGACTTCG-3', Actin reverse primer- 5'-GTTGGCATAGAGGTCTTT ACGG-3'	IDT oligo
C1566S USP9X active site mutant oligonucleotides: USP9X_FP: 5'-GAATCACAGAATTCATGTAACTAGTAGCACCGGCATTTTTCAG-3' and USP9X_RP: 5'-CTGAAAAATGCCGGTGCTACTAGTTACATGAATTCTGTGATTC-3'.	IDT oligo
C985S MIB1 active site mutant oligonucleotides: MIB1_FP: 5'-TCATGCGGTCTCCACTGAGTTGACAGGTTCC-3' and MIB1_RP: 5'-GGAACCTGTCAACTCAGTGGAGACCGCATGA-3'	IDT oligo
Primers for 1-352 TDRD3 and 1-588 TDRD3: TDRD3_F- 5'-CGCCATGGCCCAGGTGGCCGGCGCG-3', TDRD3(352) _R- 5'-CGCGTAGATCTTATTCGCCCCCTGCC-3' and TDRD3(588) _R- 5'-CGCGTACCAATGAAACTATTACTTCG-3'	IDT oligo
cDNA for human TDRD3 (1-171) was PCR amplified with primers 5'-CAGAATTCGCCACCATGGCCCAGGTGGCCGGCGCGG-3' and 5'-CAGCGGCCGCATTGTGTTTTGATAAGCTTCTC-3'	IDT oligo
