# Supplementary material for: The TDRD3-USP9X complex and MIB1 regulate TOP3B homeostasis and prevent deleterious TOP3B cleavage complexes
Source: Nat Commun. 2023 Nov 18;14:7524. doi: 10.1038/s41467-023-43151-z (PMC10657456; doi:10.1038/s41467-023-43151-z)
Supplement: Supplementary file 3 — Reporting Summary [file 41467_2023_43151_MOESM3_ESM.pdf]

nature portfolio

Corresponding author(s): Yves Pommier

Last updated by author(s): Oct 30, 2023

Reporting Summary

Nature Portfolio wishes to improve the reproducibility of the work that we publish. This form provides structure for consistency and transparency in reporting. For further information on Nature Portfolio policies, see our Editorial Policies and the Editorial Policy Checklist.

Statistics

For all statistical analyses, confirm that the following items are present in the figure legend, table legend, main text, or Methods section.

n/a	Confirmed	
☐	☒	The exact sample size (*n*) for each experimental group/condition, given as a discrete number and unit of measurement
☐	☒	A statement on whether measurements were taken from distinct samples or whether the same sample was measured repeatedly
☐	☒	The statistical test(s) used AND whether they are one- or two-sided<br>*Only common tests should be described solely by name; describe more complex techniques in the Methods section.*
☒	☐	A description of all covariates tested
☒	☐	A description of any assumptions or corrections, such as tests of normality and adjustment for multiple comparisons
☐	☒	A full description of the statistical parameters including central tendency (e.g. means) or other basic estimates (e.g. regression coefficient) AND variation (e.g. standard deviation) or associated estimates of uncertainty (e.g. confidence intervals)
☐	☒	For null hypothesis testing, the test statistic (e.g. *F*, *t*, *r*) with confidence intervals, effect sizes, degrees of freedom and *P* value noted<br>*Give P values as exact values whenever suitable.*
☒	☐	For Bayesian analysis, information on the choice of priors and Markov chain Monte Carlo settings
☒	☐	For hierarchical and complex designs, identification of the appropriate level for tests and full reporting of outcomes
☒	☐	Estimates of effect sizes (e.g. Cohen's *d*, Pearson's *r*), indicating how they were calculated

Our web collection on statistics for biologists contains articles on many of the points above.

Software and code

Policy information about availability of computer code

Data collection	BioRad ChemiDoc MP Imaging System
Data analysis	ImageJ (Fiji, 2020) ; Graph Pad Prism 9.0

For manuscripts utilizing custom algorithms or software that are central to the research but not yet described in published literature, software must be made available to editors and reviewers. We strongly encourage code deposition in a community repository (e.g. GitHub). See the Nature Portfolio guidelines for submitting code & software for further information.

Data

Policy information about availability of data

All manuscripts must include a data availability statement. This statement should provide the following information, where applicable:

- Accession codes, unique identifiers, or web links for publicly available datasets
- A description of any restrictions on data availability
- For clinical datasets or third party data, please ensure that the statement adheres to our policy

All unique/stable reagents generated in this study are available from the Lead Contact with a completed Materials Transfer Agreement. Original imaging data data generated in this study are provided with this paper as a Source Data file. Any additional information required to reanalyze the data reported in this paper is available from the lead contact upon request. Further information and requests for resources and reagents should be directed to and will be fulfilled by Lead Contact Yves Pommier (pommier@nih.gov).

Human research participants

Policy information about studies involving human research participants and Sex and Gender in Research.

Reporting on sex and gender	N/A
Population characteristics	N/A
Recruitment	N/A
Ethics oversight	N/A

Note that full information on the approval of the study protocol must also be provided in the manuscript.

Field-specific reporting

Please select the one below that is the best fit for your research. If you are not sure, read the appropriate sections before making your selection.

☒ Life sciences ☐ Behavioural & social sciences ☐ Ecological, evolutionary & environmental sciences

For a reference copy of the document with all sections, see nature.com/documents/nr-reporting-summary-flat.pdf

Life sciences study design

All studies must disclose on these points even when the disclosure is negative.

Sample size	Each experiment was repeated 3 times independently (if not mentioned otherwise in figure legends) with similar results for all figures. Sample size was determined to ensure statistical analyses. No statistical methods were used to predetermine sample size, which were chosen based on previous experience with these type of experiments in previous studies (PMID 37353483, PMID: 37024461, PMID: 34408146, PMID: 33378676, PMID: 35830799).
Data exclusions	No data exclusions.
Replication	All the experiments performed in this study were repeated either 2 or 3 times as indicated in the figure legends. All attempts at replication were successful.
Randomization	The experiments were not randomized because this work is not a randomized study on drug efficacy and safety in animal models or human. We used certain cell lines only for all the experiments and in each experiment, different cell samples started from similar conditions and treatments were allocated in controlled and deliberate manners to study the mechanisms of TOP3B-TDRD3 coupling at molecular levels.
Blinding	The investigators were not blinded to allocation during experiment and outcome assessment. Blinding was not relevant for our study because analyses were analyst independent.

Reporting for specific materials, systems and methods

We require information from authors about some types of materials, experimental systems and methods used in many studies. Here, indicate whether each material, system or method listed is relevant to your study. If you are not sure if a list item applies to your research, read the appropriate section before selecting a response.

Materials & experimental systems

n/a	Involved in the study	
☐	☒	Antibodies
☐	☒	Eukaryotic cell lines
☒	☐	Palaeontology and archaeology
☒	☐	Animals and other organisms
☒	☐	Clinical data
☒	☐	Dual use research of concern

Methods

n/a	Involved in the study	
☒	☐	ChIP-seq
☒	☐	Flow cytometry
☒	☐	MRI-based neuroimaging

Antibodies

Antibodies used	1:1000 for Mouse monoclonal anti-FLAG M2 (Millipore Sigma, St. Louis, MO, CAT#: F1804),

Antibodies used

1:10000 for Rabbit monoclonal anti-GAPDH (Cell Signaling Technology, Danvers, MA, CAT#: 2118S),
1:1000 for Rabbit monoclonal anti-HA (Cell Signaling Technology, Danvers, MA, CAT#: 3724S),
1:1000 for Rabbit monoclonal anti-TDRD3 (Cell Signaling Technology, Danvers, MA, CAT#: 5942S),
1:1000 for Mouse monoclonal anti-MIB1 antibody (B9, Santa Cruz Biotechnology, Santa Cruz, CA, CAT#: sc-393811),
1:1000 for Rabbit monoclonal anti-TOP3B antibody (abcam, Waltham, MA, CAT#: ab183520),
1:1000 for Rabbit monoclonal anti-USP9X antibody (Cell Signaling Technology, Danvers, MA, CAT#: 14898S),
1:2000 for Mouse monoclonal anti-phospho (S139)-H2AX (JBW301, Millipore Sigma, St. Louis, MO, CAT#: 05-636),
1:1000 for Mouse monoclonal Anti-PAR Polymer Monoclonal Antibody (R&D Systems, Inc. a Bio-Techne Brand, CAT#: 4335-MC-100),
1:1000 for Ub (P4D1) antibody, Cat# sc-8017, Santa Cruz Biotechnology
1:1000 for Anti-DNA-RNA Hybrid Antibody, clone S9.6, Cat# MABE1095, Millipore Sigma
1:1000 for Rabbit polyclonal TRIM41 antibody, (Cat# ab111580, Abcam)

The following secondary antibodies were used in this study:

anti-mouse IgG ECL, HRP conjugated (dilution 1:10000, GE Healthcare, Cat# NA9310)
anti-rabbit IgG ECL, HRP conjugated (dilution 1:10000, GE Healthcare, Cat# NA9340)

Validation

No in-house antibodies were used in this study. All commercial antibodies were validated by suppliers and previous references. Antibodies were further validated by siRNA transfection, as indicated below:

anti-FLAG: https://www.sigmaaldrich.com/US/en/product/sigma/f1804
anti-GAPDH: https://www.cellsignal.com/products/primary-antibodies/gapdh-14c10-rabbit-mab/2118
anti-HA: https://www.cellsignal.com/products/primary-antibodies/ha-tag-c29f4-rabbit-mab/3724
anti-TDRD3: https://www.cellsignal.com/products/primary-antibodies/tdrd3-d3o2g-rabbit-mab/5942
anti-MIB1: https://www.scbt.com/p/mib1-antibody-b-9
anti-TOP3B: https://www.abcam.com/products/primary-antibodies/top3b-antibody-ep7779-c-terminal-ab183520.html
anti-USP9X: https://www.cellsignal.com/products/primary-antibodies/usp9x-d4y7w-rabbit-mab/14898
anti-phospho (S139)-H2AX: https://www.emdmillipore.com/US/en/product/Anti-phospho-Histone-H2A.X-Ser139-Antibody-clone-JBW301,MM_NF-05-636
anti-PAR: https://www.bio-techne.com/p/antibodies/par-padpr-antibody-10ha_4335-mc-100
anti-Ub: https://www.scbt.com/p/ubiquitin-antibody-p4d1
Anti-DNA-RNA Hybrid Antibody, clone S9.6: https://www.emdmillipore.com/US/en/product/Anti-DNA-RNA-Hybrid-Antibody-clone-S9.6,MM_NF-MABE1095
anti-TRIM41: https://www.abcam.com/products/primary-antibodies/trim41-antibody-ab111580.html

Eukaryotic cell lines

Policy information about cell lines and Sex and Gender in Research

Cell line source(s)	HCT116 cells were obtained from the NCI Developmental Therapeutics Program and HEK293 cells from ATCC, Manassas, VA
Authentication	Cell line authentication was carried out using short tandem repeat analysis at Frederick National Laboratory, NCI-NIH.
Mycoplasma contamination	Cells were routinely tested for mycoplasma by MicoAlert (Lonza) and found negative.
Commonly misidentified lines (See ICLAC register)	No commonly misidentified cell lines was used.
